# Repeat-Induced Point Mutations Drive Divergence between *Fusarium circinatum* and Its Close Relatives

**DOI:** 10.3390/pathogens8040298

**Published:** 2019-12-14

**Authors:** Stephanie van Wyk, Brenda D. Wingfield, Lieschen De Vos, Nicolaas A. van der Merwe, Quentin C. Santana, Emma T. Steenkamp

**Affiliations:** Department of Biochemistry, Genetics and Microbiology, Forestry and Agricultural Biotechnology Institute (FABI), University of Pretoria, Private Bag X20, Pretoria 0028, South Africabrenda.wingfield@fabi.up.ac.za (B.D.W.); lbahlman@fabi.up.ac.za (L.D.V.); albe.vdmerwe@up.ac.za (N.A.v.d.M.); quentin.santana@fabi.up.ac.za (Q.C.S.)

**Keywords:** *Fusarium circinatum*, lineage divergence, RIP, Repeat-Induced Point mutations, segregation distortion, genome evolution

## Abstract

The Repeat-Induced Point (RIP) mutation pathway is a fungal-specific genome defense mechanism that counteracts the deleterious effects of transposable elements. This pathway permanently mutates its target sequences by introducing cytosine to thymine transitions. We investigated the genome-wide occurrence of RIP in the pitch canker pathogen, *Fusarium circinatum*, and its close relatives in the *Fusarium fujikuroi* species complex (FFSC). Our results showed that the examined fungi all exhibited hallmarks of RIP, but that they differed in terms of the extent to which their genomes were affected by this pathway. RIP mutations constituted a large proportion of all the FFSC genomes, including both core and dispensable chromosomes, although the latter were generally more extensively affected by RIP. Large RIP-affected genomic regions were also much more gene sparse than the rest of the genome. Our data further showed that RIP-directed sequence diversification increased the variability between homologous regions of related species, and that RIP-affected regions can interfere with homologous recombination during meiosis, thereby contributing to post-mating segregation distortion. Taken together, these findings suggest that RIP can drive the independent divergence of chromosomes, alter chromosome architecture, and contribute to the divergence among *F. circinatum* and other members of this economically important group of fungi.

## 1. Introduction

The genus *Fusarium* includes a diverse assemblage of fungi—many of which are plant, animal and human pathogens [1]. They also produce a wide range of highly toxic secondary metabolites that may contaminate food and feed-stocks. Because of their importance in forestry, agriculture and medicine, the genomes of many *Fusarium* species have been determined [2]. For example, the causal agent of pine pitch canker, *Fusarium circinatum*, represents one of the most important fungal pathogens of wild and planted conifers, particularly pine species [3] and its genome has not only been sequenced, but also assembled into chromosome-sized scaffolds [4,5]. This is also true for various of the close relatives of the pathogen [6,7,8]. The availability of these sequences represents an invaluable resource for understanding the factors that drive genome evolution, lineage divergence and ultimately speciation in these and other fungi. It is thus not surprising that *Fusarium* is emerging as a model for research in the field of fungal evolutionary biology [2,8]. 

Among the various *Fusarium* species complexes [9], the evolutionary history of the *Fusarium fujikuroi* species complex (FFSC) is particularly well studied [10,11]. It includes *F. circinatum* and numerous economically important pathogens of tree, fruit and cereal crops [1,12] and is estimated to have emerged during the late Miocene period, about 8.8 million years ago [11]. Since then, the FFSC has diverged into three distinct lineages or clades, which were initially thought to have Gondwanan origins and named after the geographic origin (i.e., “African”, “Asian” and “American”) of the plants from which individual species were isolated [10]. However, it is now widely accepted that the three clades have much more recent origins, which likely involved long-distance and transoceanic dispersal among continents [11]. These more recent origins are also congruent with the high levels of similarity and macrosynteny observed among the genomes of FFSC species [7,13].

One potential source of genetic variation that drives genome evolution is transposable elements (TEs) [14,15]. TEs facilitate ectopic recombination, inversions, duplications and deletions and, in extreme cases, can also cause double-stranded DNA breaks that compromise genome integrity [15,16]. For combatting TE activity, certain fungi employ defense mechanisms such as the Repeat-Induced Point (RIP) mutation pathway [17]. Since its initial description in *Neurospora crassa*, RIP has been reported in a range of ascomycetes and some basidiomycetes [18,19,20,21]. RIP is a homology-dependent mechanism that targets and permanently mutates the cytosine of duplicated motifs, by inducing adenine and thymine biased transition mutations in the targeted sequences [18,21]. In *N. crassa*, RIP has also been shown to facilitate epigenetic silencing of the targeted regions by methylating their cytosine bases [19]. This entire process occurs during sexual reproduction, prior to karyogamy and meiosis, when two copies of the genome are still present in the dikaryotic cell [19]. 

RIP plays an important role in genome architecture and evolution because its activity facilitates the formation of long stretches of AT-rich regions that are gene-poor [22,23]. Ultimately, the process may contribute to the formation of so-called “two-speed genomes,” where RIP has been shown to enhance the variability of certain genomic regions, which includes the dispensable regions in fungi harboring these sub-genomic compartments [24,25,26]. The evolution of RIP-driven lineage-specific regions further plays an important role in the development of certain genes [22,24]. This is because RIP is a “leaky” process and its transition mutations may extend into the non-duplicated regions adjacent to RIP-targeted duplicated regions [27]. In this way, RIP mutation of single-copy genes is thought to have allowed for the diversification of effector and avirulence genes in certain fungi, thereby also impacting plant-fungus interactions [22,28,29]. 

The exact molecular mechanism underlying RIP is still under investigation and not well understood. However, in *N. crassa*, a set of genes and co-factors have been identified to be essential for the RIP process. These include two canonical genes that encode the 5-cytosine methyltransferases RID (RIP deficient) and DIM-2 (defective in methylation). The RIP-associated cofactors identified in this model fungus include HP-1 (heterochromatic protein), and DIM-3, -5, -7, -8 and -9 [20,30,31]. Given this body of knowledge, most in silico investigations of RIP in other fungi are often conducted in relation to the process and presence of these known gene products in *N. crassa* [22,23,31]. 

Contemporary studies on RIP amongst *Fusarium* lineages have focused primarily on the involvement of RIP in TE- and gene-level mutations. Mutation patterns indicative of RIP activity have been experimentally or computationally studied only in the TEs of *Fusarium graminearum* [20,32], *Fusarium poae* [2], *Fusarium oxysporum* [20], and *Fusarium solani* [33]. The first experimental evidence for RIP in the FFSC was published in the early 1990s, soon after the discovery of the RIP pathway, when it was shown that RIP interfered with post-mating segregation of experimentally introduced duplicated gene regions [34]. However, it is not known whether all FFSC fungi are indeed RIP capable, as RIP capability and the extent of RIP can differ greatly among closely related lineages [20]. Moreover, RIP has not been studied in a genome-wide context amongst this important group of fungi, despite the wide availability of high-quality genome assemblies.

The aim of the current study was to evaluate the genome-wide occurrence and extent of RIP mutations in *F. circinatum* and its relatives in the FFSC. We also wanted to know whether RIP might contribute to the divergence among these fungi. We used whole-genome sequence data for representatives of all three of the FFSC clades to address the following questions: i) Do the genomes of these fungi harbor evidence of RIP, and if so, to what extent has this pathway affected them? ii) To what extent are core and dispensable chromosomes differentially affected by RIP? iii) Are RIP mutations acquired in a lineage-specific manner? iv) Can RIP explain previous observations of post-mating segregation transmission ratio distortion (TRD) amongst hybrid progeny of the FFSC species *F. circinatum* and *Fusarium temperatum* [35]? v) At the interspecies level, to what extent is RIP associated with non-synteny of homologous chromosomes and the occurrence of pseudogenes? Overall, this study thus seeks to provide evidence for RIP in *F. circinatum* and its close relatives, and to shed light on the manner in which these mutations are acquired on a genome-wide scale in these fungi. It also highlights the important role that RIP may have in both lineage divergence and genome differentiation among these fungi.

## 2. Results

### 2.1. Identification, Characterization and Potential Functionality of RIP-Associated Proteins 

For this study the genome sequences for six FFSC species with a high level of completeness, as indicated by Benchmarking Universal Single-Copy Orthologs (BUSCO) v. 3.0.2 (https;//busco.ezlab.org), were used (Table 1). *F. circinatum* and *F. temperatum* from the “American Clade”, *F. fujikuroi* and *Fusarium mangiferae* from the “Asian Clade”, and *Fusarium nygamai* and *Fusarium verticillioides* from the “African Clade”. All six of the FFSC genomes examined contained genes that encoded homologs of the two canonical enzymes of the RIP pathway, DIM-2 and RID. Genes encoding homologs of the co-factors DIM-3, -5, -7, -8, -9 and HP-1, associated with RIP activity in *N. crassa*, were identified in each of the genomes (Table 1 and Supplementary Table S1). The eight RIP-associated genes had similar functional annotations and requisite functional domains as those described for *N. crassa* (Supplementary Table S2) [20,30,36]. Among the FFSC species, the sequences of the respective genes were highly conserved, with amino acid similarities ranging from 86% to 98% for RID to 96% to 99% for DIM-3 (Table 2). Furthermore, in *F. circinatum*, RNA-seq data provided evidence of transcription for all the genes (Supplementary Table S1, Supplementary Figure S1). Further, in *F. fujikuroi*, the genomic location of the majority of H3k9me3 markers coincided with the location of mutations resembling those associated with RIP (Supplementary Figure S2), which is similar to what has been observed for *N. crassa* [17,37]. Taken together, these results suggest that, should RIP occur in the FFSC, the process is likely mediated by the RID and the DIM-2/DIM-5 pathways [17,37].

### 2.2. Genome-Wide in Silico RIP-Analysis 

**Figure 1:**
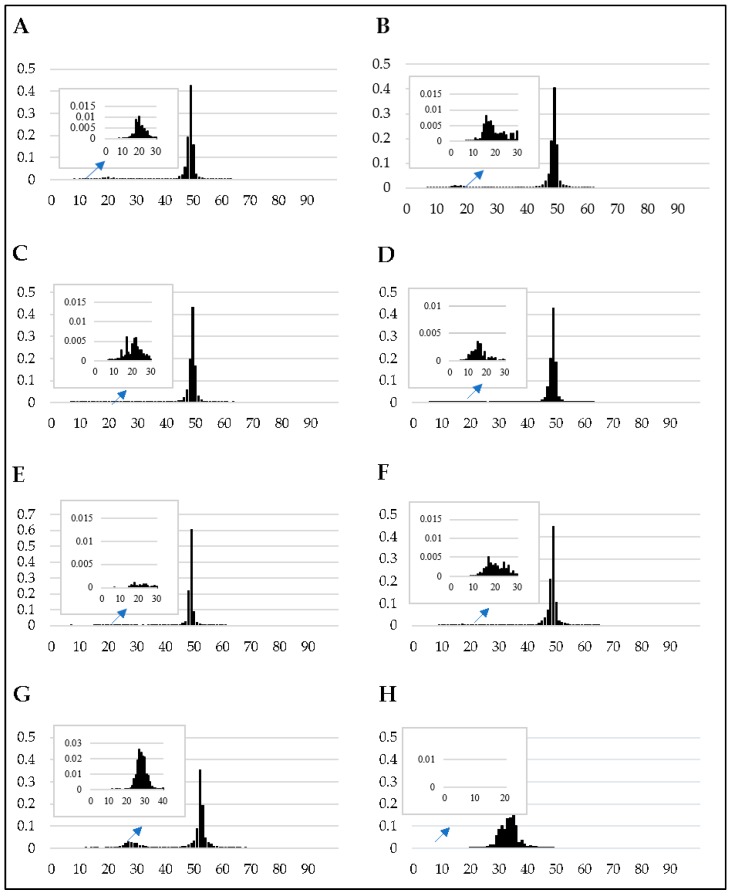
The frequency distribution of the GC content of each of the genome sequences investigated in this study (**A**–**H** represent *F. temperatum*, *F. circinatum, F. fujikuroi*, *F. mangiferae*, *F. verticillioides, F. nygamai, N. crassa*, and *C. albicans*, respectively). The x-axis indicates percentage GC content and the y-axis illustrates the proportion of the genome at a given GC content. The y-axis for the proportion of the genome containing the secondary peak has been enlarged for each of the genome assemblies investigated.

**Figure 2:**
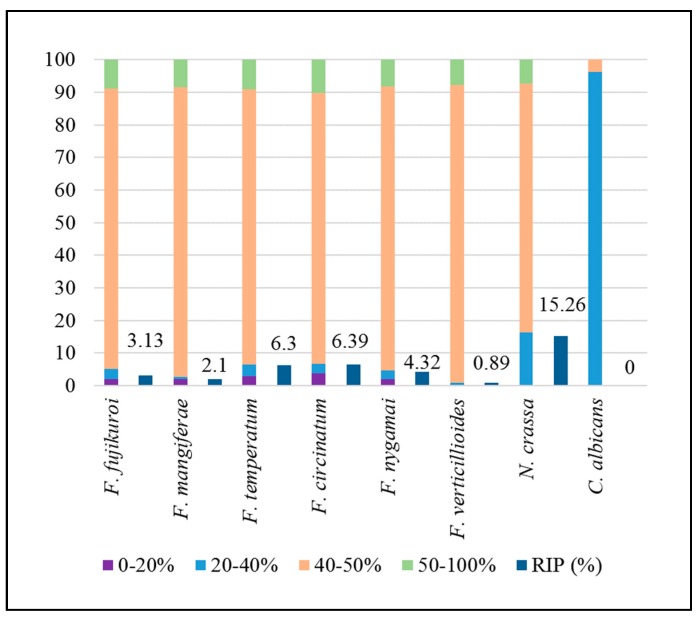
Proportions of the genomes that are characterized by each GC content range and proportions of the genomes that are affected by RIP.

As expected for RIP-competent Pezizomycotina [25], the frequency distribution of GC content across the FFSC genomes was bimodal, with the major peak at approximately 50% and a minor peak at approximately 20% (Figure 1). A similar pattern was observed in the *N. crassa* genome, while the distribution was unimodal for the *Candida albicans* genome, with a single peak at approximately 35% GC. Further, as expected for RIP-competent fungi [20,25], a substantial proportion of the FFSC genomes was GC-depleted (i.e., containing 0%–40% GC). This was particularly evident when the genomes were segmented based on specific GC content ranges (0%–20%, 20%–40%, 40%–50% and 50%–100%). An average of 2.39% and 2.5% of the total genomes of these fungi fell within the 0%–20% and 20%–40% GC content ranges, respectively (Figure 2 and Supplementary Figure S3). Within these low-GC content ranges, dinucleotide and trinucleotide frequencies were enriched for AT-rich di- and trinucleotide combinations (Supplementary Figure S3). This is in contrast to the 40%–50% and 50%–100% GC content ranges, in which the AT-rich motifs had similar frequencies to those observed for the other di- and trinucleotide combinations. 

We also detected the impact of RIP on the FFSC genomes using all three of the widely used RIP indices (Table 3 and Supplementary Table S3) [40,41,42,43]. These analyses showed that 3% to almost 10% of the various FFSC genomes contained RIP mutations for the core chromosomes (Figure 2). Additionally, in *F. circinatum*, RIP mutations generally also co-localized with TE-rich regions (Supplementary Figure S4). These data thus indicated that all of the FFSC representatives examined were likely RIP capable. 

**Figure 3:**
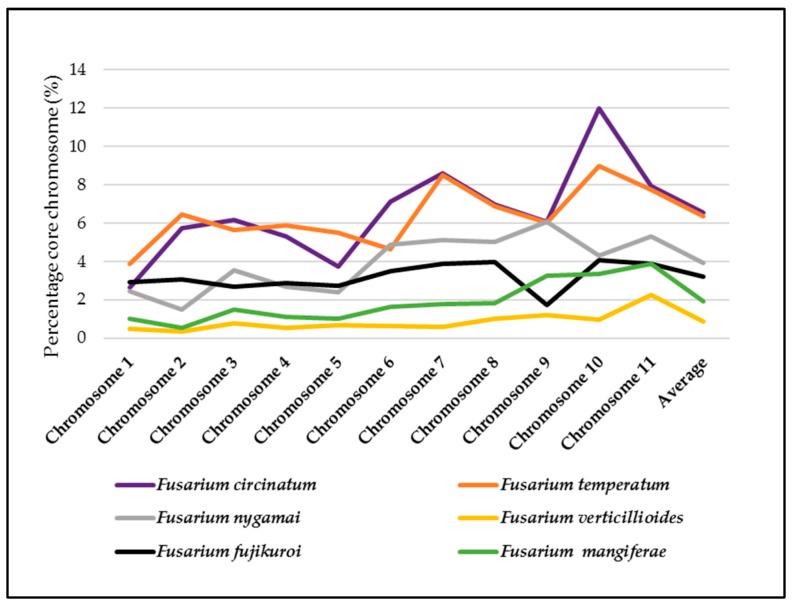
Percentage of the core chromosomes affected by RIP (y-axis). The RIP analyses were performed with a 1000 bp sliding window and a 500 bp step size for all of the core chromosomes of the different FFSC species investigated in this study.

**Figure 4:**
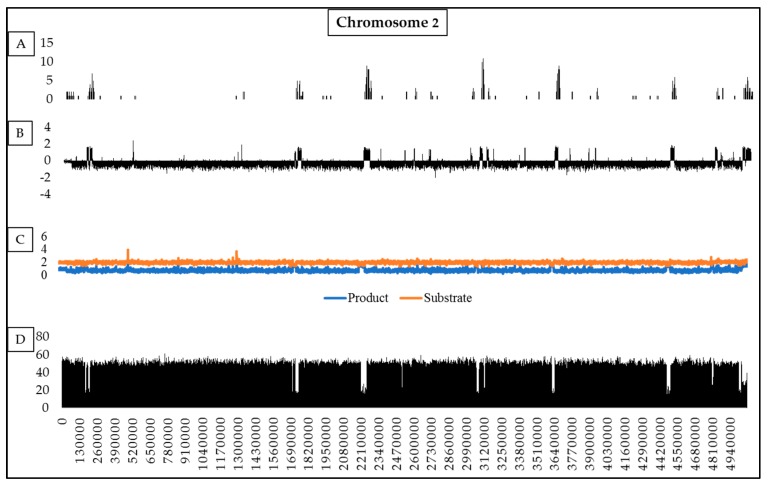
Summary of the genetic features of chromosome two of *Fusarium circinatum* (FSP34). (**A**) The distribution of TEs and repeat sequences (larger than 100 base pairs [bp]) across the length of the chromosome, (10 Kb window and 5 Kb increments). (**B**) Changes in RIP composite index values across the length of the chromosome, using a 1000 bp window and increments of 500 bp. Values above 0 indicate RIP. (**C**) Changes in RIP product (blue) and substrate (orange) index values across the length of chromosome using a 1000 bp window and increments of 500 bp. RIP product index values above 1.1 and RIP substrate index values below 0.75 indicate RIP. (**D**) Changes in GC content calculated using 1000 bp and 500 bp increments. Except for chromosome 1, similar distribution patterns for RIP index, GC content and TE incidence were also observed for the other chromosomes of this fungus (Supplementary Figure S3.1–S3.12).

For homologous sets of chromosomes (Supplementary Table S4), RIP was detected on all 11 of the core chromosomes of the fungi examined (Figure 3 and Supplementary Table S3). Our analyses showed that 0.35%–11.99% of individual chromosomes contain RIP mutations, while RIP may affect 0.86%–6.37% of the entire set of the core chromosomes in a species (Figure 3). Across individual chromosomes, changes in RIP product, substrate and composite index values also corresponded with changes in GC content, (see Supplementary Figure S3.1–S3.12). For example, on chromosome two of *F. circinatum*, low-GC regions co-occurred with those in which values for the various RIP indices were suggestive of RIP (Figure 4). 

Despite the high degree of similarity among homologous core chromosomes (Supplementary Table S4), the extent to which RIP mutations were accumulated per chromosome varied among species (Figure 3, Supplementary Table S3 and Figure S5). For example, the genomes of the two “American clade” species (*F. circinatum* and *F. temperatum*) were in many cases markedly different in terms of the impact of RIP on homologous chromosomes (e.g., respectively 11.99% and 8.97% of chromosome 10, Supplementary Figure S5). Overall, the RIP capability among the six species for the core chromosomes ranked as follows: *F*. *circinatum* > *F. temperatum* > *F. nygamai* > *F*. *fujikuroi* > *F. mangiferae* > *F. verticillioides*.

In addition to the core chromosomes, our data showed that RIP also affected the dispensable chromosomes (chromosome 12) of *F. circinatum*, *F. temperatum*, *F. nygamai, F. mangiferae* and *F. fujikuroi* (Table 4). With the exception of *F. circinatum*, all these chromosomes were more extensively affected by RIP than their corresponding core chromosomes (Table 4). Further, the size of the RIP-affected regions was markedly higher for all FFSC species with dispensable chromosome assemblies available compared to that of the *F. circinatum* dispensable chromosome. Furthermore, the GC content of these chromosomes was 41.05%–43.02%, which is much lower than the ca. 50% observed for entire FFSC genomes. However, the GC content of the dispensable chromosome of *F. circinatum* was 46.36% and the proportion of the chromosome affected by RIP was 4.85%, which is less than that observed across the entire genome of this fungus (Figure 2). 

To investigate the genomic landscape associated with RIP, we identified LRARs (large RIP-affected regions) on all chromosome sequences of the FFSC genomes examined (Table 3 and Table 4, Supplementary Table S5). LRARs were generally characterized by reduced GC content (e.g., 20.8% compared to 46.97% GC across for the total *F. circinatum* genome) and their number and size varied across chromosomes and species, but they typically accounted for a large proportion of the genomes investigated (Table 3). The average size of LRARs ranged from 6219 bp in *F. verticillioides* to 16,625 bp for *F. temperatum*, with the largest LRAR (79,000 bp) occurring on chromosome 7 of *F. fujikuroi* (Supplementary Table S5). With regards to dispensable chromosomes, those of *F. nygamai* contained 14 LRARs compared to those of *F. circinatum*, with its single LRAR. LRARs were generally also gene-sparse relative to the rest of the genome (Table 3 and Supplementary S6). For example, the gene density of the LRARs of *F. circinatum* were 1.5 genes per 100 kilo bp (Kb), while the average gene density for this fungus is 34.41 genes per 100 Kb. Based on the Blast2GO data for *F. circinatum*, a substantial proportion of the LRAR genes had no GO terms (i.e., 24/36 for *F. circinatum*), although REVIGO’s summary of those with identifiable GO terms suggested roles in diverse molecular functions and biological processes (Supplementary Table S7).

### 2.3. RIP Analysis of Regions Showing TRD

**Figure 5:**
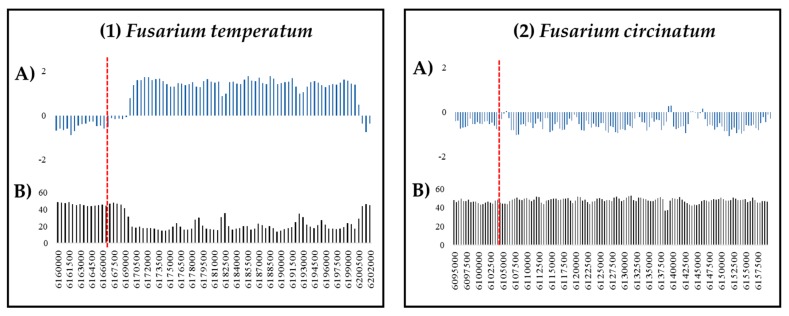
Summary of the genetic features of the genomic regions of the GA/CC355be amplified fragment length polymorphism (AFLP) marker (indicated with a red dotted line) on chromosome one of (1) *F. temperatum* and (2) *F. circinatum*. (**A**) Bar charts showing changes in RIP composite index values and %GC (**B**), respectively, across the genomic regions investigated (indicated in base pair position). RIP composite index values above 0 indicate RIP. These analyses used a 1000 bp window at 500 bp increments.

To determine whether previous observations of TRD amongst hybrid progeny of the FFSC species *F. circinatum* and *Fusarium temperatum* could have been due to RIP, we utilized a set of DNA markers that displayed post-mating segregation distortion and that were postulated to be the result of chromosomal differences between the parental species [35]. For comparative purposes, we also included markers from this previous study that segregated in a Mendelian fashion. In total, 60 markers were mapped to the genome assemblies of *F. circinatum* and *F*. *temperatum*. Analysis of the genomic regions of the respective markers showed that they were species-specific. They also differed with respect to the total size of the RIP-affected regions in which they occurred (Supplementary Tables S8 and S9). For example, the TRD marker GA/CC353be was mapped to chromosome one in *F. temperatum* and showed a corresponding homology to the same chromosomal region in *F. circinatum*. For *F. temperatum*, the marker was located adjacent to 66 consecutive RIP-positive windows (32,500 bp) as opposed to only a few in *F. circinatum* (Figure 5).

Of the 37 TRD markers identified previously [35], we were able to map 31 to the genome of *F. temperatum* and 30 to that of *F. circinatum* (Supplementary Table S8). They were distributed across seven of the eleven core chromosomes in both these genomes. For both species, the TRD markers were mostly located in regions with RIP (19/30 [63%] of *F. circinatum* markers 18/31 [58%] of *F. temperatum* markers) (Table 5). A total of 79 and 74 RIP-positive windows were located within the genomic regions associated with TRD for *F. temperatum* and *F*. *circinatum*, respectively, which accounted for 2.17% (*ca*. 79 Kb) and 2.4% (*ca*. 74 Kb) of the total RIP-affected proportion of the respective genomes. Conversely, of the 30 Mendelian markers, 26 were mapped to the genome of *F. temperatum* and 29 to that of *F. circinatum*, where they were distributed across all eleven of the core chromosomes in both species (Supplementary Table S9). Of these markers, nine (31%) and six (23%) were located in RIP-positive windows in *F. circinatum* and *F. temperatum*, respectively (Table 5). Based on a chi-square test of independence, the null expectation that the frequency of RIP mutations among the TRD markers is the same as among those inherited in a Mendelian fashion, was rejected at a 99.99% confidence level (*P* < 0.01) for both species. 

### 2.4. Pseudogenization and Loss of Homology 

To investigate possible RIP-associated pseudogenization in *F. circinatum*, all LRARs (including 10,000 bp up- and downstream directly adjacent to the LRARs) were analysed. Many more putative pseudogenes were detected within and around LRARs than in the rest of the *F. circinatum* genome (Supplementary Table S10). On average, 13.2% of genes in and around LRARs represented pseudogenes compared to 8.9% pseudogenes across the whole genome. A total of 138 putative pseudogenes (from 1046 predicted genes investigated) were identified within the 162 LRARs and neighboring regions of *F. circinatum*. Most of these lacked start (59 pseudogenes) and/or stop codons (27), or encoded short polypeptides consisting of fewer than 100 amino acids (49). A small number of the putative pseudogenes also contained multiple stop codons within a single sequence (3) or encoded peptides with less than 20 (3) or 40 (9) amino acids. 

**Figure 6:**
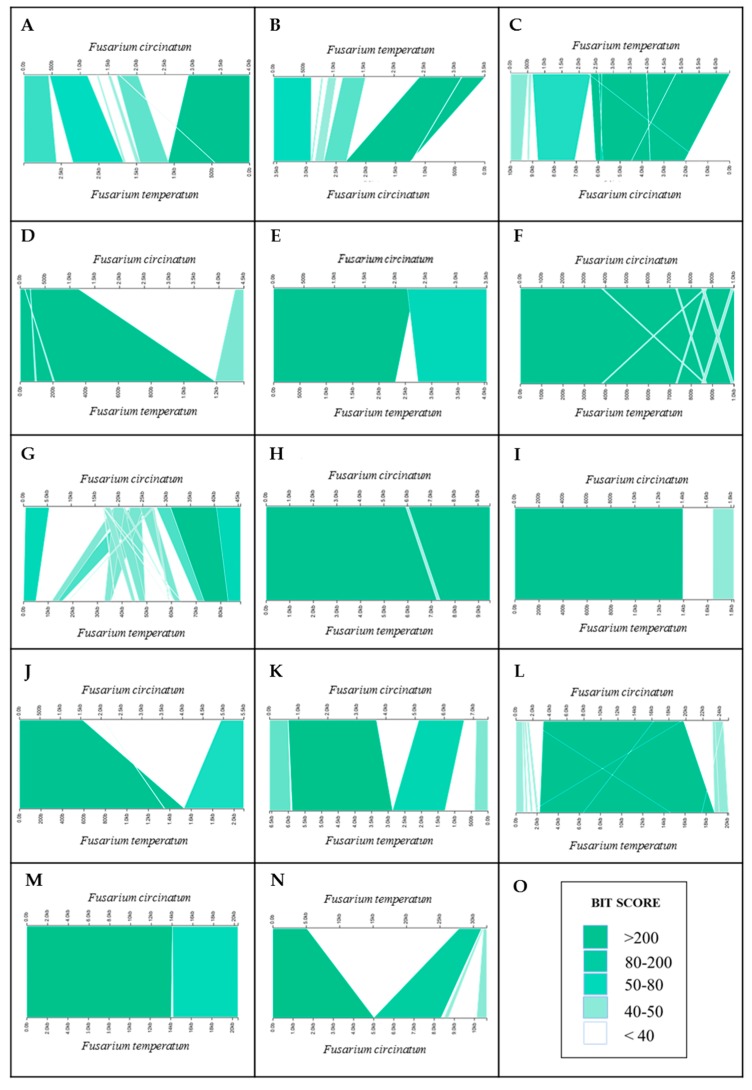
Illustration of NCBI BLASTn alignment results (Table S11) of 14 homologous genomic regions in *Fusarium circinatum* and *Fusarium temperatum* with opposite RIP statuses (Tables S8 and S9) of the transmission ratio distortion markers. Panels **A**–**N** depict alignments for the genomic regions underlying the markers AA/TC121bh, GA/AC523bh, AG/AC315bh, AA/AC255bh, AT/AC273bh, AA/AC408bh, AA/CC285bh, GA/TC291bh, AA/CC564be, AA/TC116be, CA/TG416be, GA/TC169bh, GA/TC287bh, and CA/TG413be, respectively. Panel **O** shows the color gradient used to illustrate NCBI BLASTn BIT score values (where the darkest green shade represents BIT scores above 200 and white BIT score values below 40) across the length of the alignments.

To investigate possible RIP-associated loss of homology, we analysed genomic regions with opposite RIP-statuses by specifically focusing on the Mendelian and TRD markers identified above. For *F. circinatum* and *F. temperatum*, changes in nucleotide identity across the length of the alignment of 14 homologous regions surrounding the TRD and Mendelian markers showing opposite RIP statuses (occurring in a region that is either RIP-positive or RIP-negative) were examined in order to determine whether RIP could bring about changes in homology and synteny. Although two of these regions (i.e., those associated with markers GA/TC291bh and GA/TC287bh on chromosomes one and seven in *F. circinatum* and *F. temperatum*) were fully homologous and mostly aligned well, the remaining 12 alignments showed extensive differences in nucleotide identity values, as well as changes in synteny and homology (Figure 6; Supplementary Table S11). For seven of these (i.e., markers AA/AC255bh, GA/AC523bh, AT/AC 273bh, AA/CC564be, AA/TC116be, CA/TG416be and CA/TG413be), one or both of the sequences contained substantial sequence sections that could not be aligned; e.g., only 16% of a 4.5 Kb region in *F. circinatum* could be aligned to the corresponding 1.4 Kb region in the genome of *F. temperatum* for marker AA/AC255bh, and as little as 24% of a total of 33.5 Kb in *F. temperatum* could not be aligned to the corresponding ~ 10.3 Kb region in *F. circinatum* for marker CA/TG413be. Alignments for the regions containing the remaining markers were characterized by a similar lack of alignability in addition to extensive internal rearrangements (i.e., markers AA/TC121bh, AA/AC315bh, AA/CC285be and GA/TC169bh), or mostly internal rearrangements (marker AA/AC408bh).

## 3. Discussion

### 3.1. The Genomes of F. circinatum and Its Relatives Harbor Evidence of RIP

The FFSC genomes examined here harbored the known hallmarks of RIP. We observed bimodal distributions of GC content frequencies for all six genomes, with a minor peak in the low-GC content ranges (10%–30%) and a major peak at approximately 50%. Such bimodal GC content frequency distributions are common genomic features of RIP-capable fungi [25]. Our data further indicated that the FFSC genomes consist of a GC-depleted AT-rich sub-genomic compartment and a compartment that is GC-equilibrated (i.e., the proportion of the genome that contains the high GC content ranges, 40%–50% and 50%–100%, which constitutes the majority of the genome assembly), which is also comparable to those of other RIP-capable Sordariomycetes [25]. Substantial proportions of the FFSC genomes further appeared to be RIP affected, in that they showed the expected increased frequencies of RIP product dinucleotides (TpA and ApT) and reductions in RIP substrate dinucleotides (CpA [TpG] and ApC [GpT]) [20,31]. Further, in *F. circinatum*, the location of TEs and repeated regions generally coincided with the location and frequencies of RIP mutations. These patterns thus suggest that *F. circinatum* and the other FFSC species examined are indeed RIP capable or were RIP capable until relatively recently. 

All the FFSC genomes examined in this study contain genes that putatively encode for proteins and co-factors known to be associated with RIP and RIP-directed methylation similar to that described in *N. crassa* [17,30,31,38,39]. In *F. circinatum*, we also found evidence of expression for the canonical RID and DIM-2 cytosine methyl-transferase genes, as well as those for the RIP-associated DIM-3, -5, -7, -9 and HP-1 proteins. In *F. fujikuroi*, we further found evidence of co-localization of DIM-5-directed H3k9me3 markers and RIP mutations, suggesting that DIM-2/DIM-5-mediated RIP occurs in this fungus and possibly also in the others examined. In the *F. fujikuroi* genome, the distribution of H3K9me3 markers has been associated with regions that are silenced by heterochromatin [7]. Our data thus suggest that, similar to what has been shown for *N. crassa* [38], the lysine methyltransferase activity of DIM-5 becomes induced by the occurrence of repeated sequences [44], followed by the binding of the HP-1 protein [45] that recruits the DNA methyltransferase DIM-2 to the tri-methylated lysine of histone H3. The 5-methylcytosine formed by DIM-2 then becomes deaminated to subsequently cause C to A transitions [36]. The latter 5-cytosine methylation may also be catalyzed by RID, in a pathway independent from DIM-2 [46]. Our study therefore shows that FFSC species can undergo RIP mutation through either or both the RID and DIM-2-mediated pathways and that future research should seek to functionally confirm the roles of these enzymes in *F. circinatum* and its relatives within the FFSC. 

The six genomes examined differed substantially in terms of their total genomic proportions affected by RIP. These ranged from 0.89% for the *F. verticillioides* genome to as much as 6.39% for the *F. circinatum* genome. In many cases, these RIP-affected regions occurred in long stretches ranging from 4 Kb up to as much as 79 Kb. Similar findings have been reported for fungi such as *Neurospora tetrasperma* and *Leptosphaeria maculans* [22,47]. Further, the FFSC genomes differed considerably in terms of their RIP mutations, which is similar to previous observations. For example, similar to the FFSC species, RIP dinucleotide products were readily identifiable in the *N. crassa* genome [48], while other RIP-capable fungi such as *Metarhizium robertsii* has little to no RIP products, particularly in their TE sequences [49].

One possible explanation for the variation in the total proportions of RIP-affected regions among FFSC genomes may relate to the reproductive strategies employed by these fungi. Although the FFSC can reproduce mitotically (clonally), many species in this complex, especially those examined here, are also known to have sexual stages in nature or under laboratory conditions [1]. Because the RIP pathway is exclusively active in sexually reproducing fungal populations [19], it is expected that the genomes of sexually reproducing RIP-capable species will be extensively affected by RIP. By contrast, populations that are reproducing clonally or that are asexual may lack such regions and/or may only display remnants or relics of historical RIP [20,50]. Therefore, to fully understand variations in RIP-activity, the reproductive biology and life history of a fungal isolate need to be taken into consideration. Other causes proposed to explain variations in RIP capability between related species include the extent of TE integration and gene duplication, levels of male and female fertility, and RIP enzyme activity and specificity [51].

### 3.2. RIP Can Change Genomic Environments and Gene Functionality

Analysis of RIP substrates and products on homologous chromosomes revealed that RIP mutations are acquired in an independent or unique manner in each of the respective FFSC genomes examined. For instance, locations and proportions of the core chromosomes affected by RIP differed greatly between the American clade species despite these fungi sharing a recent common ancestor. A similar trend of apparent lineage-specificity was also observed for the dispensable chromosomes of the FFSC fungi. Our data showed that those of *F. fujikuroi, F. nygamai* and *F. temperatum* were all extensively targeted by RIP and contained numerous non-homologous LRARs, compared to the *F. circinatum* dispensable chromosome that is much less affected by RIP and had a lower LRAR content. Such large differences in the density and distribution of RIP mutations suggest the RIP pathway may play an important role in the evolution and divergence of both core and dispensable chromosomes. For example, RIP on chromosome 12 of *F*. *temperatum* is considerably higher than on any of its core chromosomes. The higher levels of RIP on dispensable chromosomes may be due to their genetic composition, particularly with respect to repeat and TE content and the presence of duplicated genes that are prime targets for RIP [51,52]. Similar patterns have also been recorded in isolates of *L. maculans* [53]. However, these findings are in contrast to those reported for lineages of *F. poae* that completely lack RIP activity or RIP mutations in the TE-rich regions of its dispensable chromosomes [2,54]. Nevertheless, the extent of intra-specific variation of RIP should be investigated in order to fully understand the role of RIP on core and accessory chromosome evolution.

RIP-affected regions differ substantially in their coding content from the rest of the genome. We showed that these regions of the FFSC genomes were particularly enriched for dinucleotides TpA and ApT associated with RIP products, as well as trinucleotide sequences associated with the so-called “amber” (TAG) and “ochre” (TAA) stop codons, which is typical for RIP-capable fungi [55,56]. Accordingly, RIP mutations may disrupt coding sequences and promote the development of premature stop codons, leading to the formation of pseudogenes and ultimately hindering the formation of functional gene products [22,32,56]. In most cases, this pseudogenization process is not limited to RIP-affected regions, because the inherent leakiness of RIP [27,55,56] allows for the formation of pseudogenes in regions adjacent to or near RIP-targeted areas [22]. Similar trends were also observed in the genome of *F. circinatum* where RIP-affected regions were gene-sparse with LRARs and their flanking regions containing numerous putative pseudogenes. Therefore, as has been shown in *N. crassa, N. tetrasperma* and *L. maculans* [22,25,47], RIP also appears to drive the formation of AT-rich genomic regions in the FFSC that ultimately impacts the gene content of the targeted regions.

Apart from promoting pseudogenization, RIP activity may also affect the overall biology of a fungus. In *L*. *maculans*, RIP-driven pseudogenization of effector and avirulence genes has contributed to the proliferation of virulent strains [56]. These strains typically have truncated avirulence genes because they were subjected to TE integration and deactivation by RIP, and their subsequent non-functionality has allowed these strains to avoid host detection [56]. Further, RIP-associated pseudogenization and gene-level differentiation facilitates the evolution of shortened gene products, particularly those that are secreted [22,25]. RIP-driven sequence diversification may further promote the formation of premature stop codons in longer protein sequences, producing novel, shortened secreted proteins in a species-specific manner. The evolution of shortened secreted protein products through RIP-directed mutagenesis has played a fundamental role in the development of a parasitic lifestyle amongst nematode-trapping fungi [24]. In this way, the RIP pathway may also influence various aspects of the lifestyle, host range and pathogenicity of fungi in the FFSC.

### 3.3. RIP May Contribute to Lineage-Divergence 

As initially observed in the late 1980s for *N. crassa* [19], RIP-associated non-Mendelian inheritance of certain loci may be common among fungi [34]. In the early 1990s, this phenomenon was also demonstrated for the first time in the FFSC for *F. verticillioides,* where experimentally introduced duplicated regions were not inherited in a Mendelian fashion and displayed TRD [19,34]. In the current study, we showed that most of the loci exhibiting TRD in an interspecific cross in *F circinatum* and *F. temperatum* were located within or in the vicinity of RIP-affected regions, suggesting that RIP may hinder recombination during meiosis, thus causing deviation from the expected Mendelian ratios of segregation in hybrid progeny of a cross between these species. 

Although clear explanations for the TRD observed in *F. verticillioides* were not apparent at the time [34], our results suggest that it may, in part, be due to RIP-associated processes. All of the FFSC genomes examined contained independently acquired stretches of DNA bearing the hallmarks of RIP. Such accumulations of RIP mutations may enhance sequence variability among fungi, and together with RIP-directed heterochromatin formation, and RIP-directed methylation may possibly cause reduced recombination frequencies in the regions affected [42,57,58]. In turn, reductions in recombination frequencies facilitate the independent evolution of the affected regions. For example, in *N. tetrasperma*, suppressed recombination at the mating-type (MAT) locus has affected a large proportion of the chromosome harboring it [47]. In fact, the accumulation of RIP mutations adjacent to the MAT locus, along with suppressed recombination and concomitant reductions in gene flow, might have driven the formation of the unusual architecture of *N. tetrasperma*’s MAT-containing chromosome [47]. This chromosome is mostly idiomorphic in that approximately 75% of it evolves in a mating-type specific manner [59,60,61]. It is, therefore, possible that similar RIP-associated processes may drive the divergence of various regions in the genomes of FFSC species. 

To address the notion that RIP-associated processes may drive divergence among the genomes examined, we compared the sequences of loci with opposite RIP profiles. In most cases, these analyses revealed loss of both homology and synteny in genomic regions displaying opposite RIP statuses. Similar to what has been shown for the *N. tetrasperma* MAT-chromosome, these regions in the FFSC genomes are thus likely the product of processes associated with RIP. In other words, these lineage-specific regions are formed when RIP decreases sequence similarity in the targeted regions, while RIP-directed methylation and limited sequence homology leads to the suppression of recombination that, under normal circumstances, would have eliminated or reduced sequence variability. The accumulation of such independently evolving regions may ultimately drive divergence of entire genomes. Overall, these processes may also be crucial for the formation and maintenance of species boundaries [62]. The whole divergence process can be enhanced even further by a range of additional factors; e.g., chromosome loss or large genomic rearrangements can interfere with complementary base pairing during homologous recombination [63], while geographical isolation becomes a physical barrier to gene flow [64]. Other large inter-chromosomal inversions, such as those observed among American clade species of the FFSC [5,14,65], likely also hamper recombination and gene flow between populations. 

In summary, the availability of whole-genome sequences assembled into chromosome-sized scaffolds have allowed for greater insight into the role RIP plays on a genome-wide scale in the FFSC. Our data suggest that these fungi are indeed RIP capable and that the process has affected large parts of their genomes. We also showed that RIP mutations are acquired uniquely in a lineage-specific fashion and that such regions can represent a considerable proportion of both core and dispensable chromosomes. RIP also drives the independent divergence of chromosomes and alters chromosome architecture, while RIP and the processes associated with it likely play key roles in the evolution of this important group of fungi.

## 4. Materials and Methods 

### 4.1. Genome Sequences

The genome sequences for eight fungi were used in this study (Table 1). These included six representatives of the FFSC for which chromosome-sized scaffolds were available, i.e., *F. circinatum* and *F. temperatum* from the “American Clade”, *F. fujikuroi* and *F. mangiferae* from the “Asian Clade”, and *F. nygamai* and *F. verticillioides* from the “African Clade”. The completeness of the six FFSC genome assemblies was verified with the Benchmarking Universal Single-Copy Orthologs (BUSCO) v. 3.0.2 tool using the “Sordariomyceta” dataset [66] (Table 1). Where relevant, we also included the genome sequences for *N. crassa* and *Candida albicans*, which are, respectively, RIP capable and RIP incapable [20,43], as positive and negative controls. 

Genome sequences, including those for core and dispensable chromosomes, were obtained from the database of the National Centre for Biotechnology Information (NCBI). Where needed for the respective *Fusarium* genomes, assemblies were annotated using WebAUGUSTUS [67] by using the gene models for *F. graminearum*. For the *F. circinatum* genome, TEs and repetitive regions were annotated with REPET v. 2.5 [68].

### 4.2. Identification, Characterization and Potential Functionality of RIP-Associated proteins

Nucleotide and protein BLAST searches were used to identify the genomic position of genes encoding proteins involved in the RIP pathway. These included genes encoding HP-1, RID and DIM--2, -3, -5, -7, -8 and -9 [20,30,31]. FFSC genes with homology (Expect-value < 1 × 10^−5^) to those described in *N. crassa* were identified using the CLC Genomics Workbench v. 8.0 (CLCbio, Aarhus, Denmark). Their functional annotations, predicted functional domains, protein family (PFAM) membership and Gene Ontology (GO) were determined using InterProScan v. 5 [69]. Similarity among the respective sets of RIP protein sequences was estimated with the CLC Genomics Workbench following multiple alignments with MAFFT v. 7 [70]. To determine whether these genes are expressed in *F. circinatum*, their sequences were compared to the RNA-seq data. RNA was extracted from one-week old *F. circinatum* (FSP34) isolate grown on half-strength potato dextrose broth at 25 °C, using a RNeasy Plant mini kit (QIAGEN, N.V., Hilden, Germany) according to the manufacturer’s specifications. RNA-seq libraries (Ion Xpress, RNA-seq library preparation kit, Thermo Fisher Scientific, Applied Biosystems, Invitrogen, USA) were sequenced (Ion Torrent, NGS) at the University of Stellenbosch, South Africa (unpublished RNA-seq data; Ms. M. Phasha, Forestry and Agricultural Biotechnology Institute, [FABI], University of Pretoria). RNA-seq data were analyzed using the CLC Genomics Workbench. A gene was considered expressed if it had a reads per kilobase per million mapped reads (RPKM) value ≥ 0.2 and at least 3 unique gene reads mapping to it [71].

In order to gain further insight on the functionality of RIP-associated gene products, we also utilized the previously determined genomic positions of H3k9me3 markers (i.e., positions bound to histone H3 that were trimethylated at lysine 9) in *F*. *fujikuroi* [7]. These markers are known to co-localize with RIP mutations in *N. crassa* due to the actions of, amongst others, HP-1, the DNA methyltransferase DIM-2 and lysine methyltransferase DIM-5 [42]. The *F*. *fujikuroi* genome assembly and corresponding H3K9me3 marker positions determined previously [7] and compared to those in which RIP products and substrates were detected using whole genome in silico analysis (see below). 

### 4.3. Genome-Wide in Silico RIP-Analysis 

Homologous chromosome-sized scaffolds of the various FFSC genome assemblies were identified using LASTZ alignments v. 1.02.00 [72] in Geneious R8 [73]. The *F. circinatum* chromosome assembly was used as a reference. Homologous chromosome sets were then subjected to RIP analysis using The RIPper [43]. RIP indices were calculated for all sequences, using a 1000 base pair (bp) sliding window and 500 bp step. These analyses also utilized stringent parameters (i.e., RIP product index > 1.1, RIP substrate index ≤ 0.75 and RIP composite index > 0) to limit false positives (i.e., regions erroneously suggested to contain RIP mutations), as suggested previously for these and other Ascomycota [43].

Changes in gene and GC content, and mono-, di-, and tri-nucleotide frequencies were further determined for each FFSC genome assembly investigated. Gene density and GC content were determined with the CLC Genomics Workbench. Mono-, di- and tri-nucleotide frequencies were calculated using the Jensen–Shannon divergence statistic implemented in OcculterCut (v. 1.1) [25]. OcculterCut was further used to identify AT-rich regions in each respective FFSC genome sequence. For comparative purposes, all these analyses were further performed on the genome sequences of *C. albicans* and *N. crassa*.

To investigate the potential changes brought about by RIP to the genomic landscape of each species, large RIP-affected regions (LRARs) were identified using the ‘calculate LRAR’ tool implemented in The RIPper [43]. Default parameters were used for defining LRARs, i.e., genomic regions spanning at least 4000 bp or seven consecutive RIP-affected windows [43]. For these LRARs, we then investigated changes in gene density. The predicted functions of the genes encoded in LRARs on the core and dispensable chromosomes of *F. circinatum* were determined using Blast2GO [74]. The identified GO terms were summarized using REVIGO [75].

### 4.4. RIP Analysis of Regions Showing TRD

A set of amplified fragment length polymorphism (AFLP) markers, previously demonstrated to experience TRD, were used in this study [35]. These markers were developed for a genetic linkage map of an interspecific cross between *F. circinatum* and *F. temperatum* [76]. Of the 252 markers reported, 37 markers showed TRD at the 0.1% significance level (α = 99.9%) [35]. In the current study, we determined the location of these TRD markers on the genomes of *F. circinatum* and *F. temperatum* by making use of an in silico approach that was described previously [14]. The sequences containing TRD markers and the regions flanking them (10,000 bp immediately up- and downstream of the marker) were then subjected to RIP analysis as described before. For comparative purposes, a set of 31 AFLP markers that showed inheritance according to expected Mendelian ratios (1:1 because *F. circinatum* and *F. temperatum* are haploid organisms) were investigated in the same way. These were randomly selected from those reported [76] using Randomizer [77]. A chi-square test was used to evaluate whether the RIP status (i.e., RIP affected or not affected by RIP) of the two sets of markers differed significantly (*P* < 0.05), where the null expectation was that the frequency of RIP among the TRD markers is the same as among those inherited in a Mendelian fashion. 

### 4.5. Pseudogenization and Loss of Homology 

For *F. circinatum*, all LRARs and their proximal genomic regions (i.e., 10,000 bp up- and downstream directly adjacent to the LRARs) were investigated for the presence of putative pseudogenes. This was achieved by reannotating the genome with WebAUGUSTUS as described above, to retrieve annotations for complete, as well as partial or truncated genes. Within these sequences, the CLC Genomics Workbench was then used to identify putative pseudogenes based on the presence of premature stop codons or multiple in-frame stop codons, as well as the loss of a start codon that may cause non- or partially functional proteins.

Loss of homology due to RIP was investigated in genomic regions that harbored markers with opposite RIP statuses. For this purpose, the identified Mendelian and TRD markers were used. Homologous regions were identified with BLAST, implemented in CLC genomics and aligned using NCBI BLASTn (Expect-value < 1 × 10^−5^). Where homologous regions could not be identified, the search included neighboring genomic regions as described before. The results were visualized with Kablamo v. 1.0 [78] using the XML file output retrieved from NCBI alignments analyses.

## Figures and Tables

**Table 1:** Accession numbers of genome sequences used in this study.

Species	Strain	Accession Number ^1^	Percentage Completeness ^2^
*Fusarium circinatum*	FSP34	AYJV00000000.2	98.1
*Fusarium temperatum*	Netza 9	LJGR00000000.1	98.8
*Fusarium nygamai*	MRC 8546	LBNR00000000.1	98.7
*Fusarium fujikuroi*	IMI 58289	GCA_900079805.1	98.6
*Fusarium mangiferae*	MRC 7560	FCQH00000000.1	99.0
*Fusarium verticillioides*	7600	AAIM00000000.2	98.3
*Neurospora crassa*	OR74A	AABX00000000.3	98.1
*Candida albicans*	SC5314	GCA_000182965.3	97.9

^1^ All genomes were obtained from the National Centre for Biotechnology Information (NCBI) database (https://www.ncbi.nlm.nih.gov). ^2^ See Supplementary Table S4B.

**Table 2:** Proteins known to be involved in RIP and the genes encoding them in the *Fusarium* genomes investigated here.

Protein Name ^1^	Accession Number ^2^	Gene Identifier (% Similarity to the *Neurospora crassa homolog*) ^3^						
		*Fc*	*Ft*	*Ff*	*Fm*	*Fv*	*Fn*	Within FFSC % similarity
HP-1	XP_957632.1	g 30.102 (59.89%)	g 30.82 (59.32%)	FF_062931 (61.80%)	FMAN_09299 (61.80%)	FVEG_013876 (59.89%)	g 3888.1 (58.54%)	90.58%–98.39%
RID	XP_011392925.1	g 24.18 (36.08%)	g 25.67 (35.29%)	FF_06164 (36.27%)	FMAN_09165 (42.36%)	FVEG_02018 (35.4%)	g 3766 (35.22%)	86.10%–97.68%
DIM-2	XP_959891.1	g 22.31 (48.8%)	g 23.9 (48.83%)	FF_08441 (47.1%)	FMAN_10524 (48.68%)	FVEG_11429 (49.44%)	g 4731.1 (50.07%)	90.39%–97.78%
DIM-3	XP_960652.1	g 17.69 (89.07%)	g 16.59 (88.58%)	FF_02702.1 (91.49%)	FMAN_04997 (91.48%)	FVEG_08024 (91.49%)	g 4826t1 (88.58%)	96.27%–99.56%
DIM-5	XP_957479.2	g 28.0 (69.36%)	g 29.67 (61.27%)	FF_07620.1 (62.43%)	FMAN_07768 (61.56%)	FVEG_08911 (61.88%)	g 2471.1 (62.43%)	95.93%–97.97%
DIM-7	XP_961308.2	g 14.27 (28.87%)	g 14.74 (36.62%)	FF_02137.1 (34.36%)	FMAN_04175 (30.43%)	FVEG_07938 (35.26%)	g 4746.1 (27.05%)	87.23%–96.12%
DIM-8	XP_962347.1	g 13.103 (52.63%)	g 14.50 (53.12%)	FF_04892.1 (53.02%)	FMAN_02500 (42.55%)	FVEG_06222 (53.64%)	g 1063.1 (53.39%)	95.95%–97.72%
DIM-9	XP_956278.3	g 15.26 (74.74%)	g 15.10 (75.79%)	FF_0162701 (75.79%)	FMAN_00488 (77.89%)	FVEG_09499 (76.84%)	g 31951 (76.84%)	87.77%–93.87%

^1^ Genes needed for Repeat-Induced Point (RIP) and RIP-associated methylation [30,38,39]. ^2^ NCBI accession numbers for the proteins encoded by *N. crassa*. ^3^ *Fc*, *Ft*, *Ff*, *Fm*, *Fv* and *Fn* refer to *F. circinatum*, *F. temperatum*, *F. fujikuroi*, *F. mangiferae*, *F. verticillioides* and *F. nygamai*, respectively. The genomic location of homologous *Fusarium* sequences was determined using BLASTp (Protein Basic Local Alignment Search Tool) searches with the *N. crassa* genes against the respective genome assemblies (see Supplementary Table S1 for details regarding chromosome/scaffold/contig/supercontig locations). Percentage amino acid identity with the respective *N. crassa* sequences was determined using BLASTp searches and is indicated in brackets.

**Table 3:** RIP statistics of the core chromosome assemblies for six fungi in the *F. fujikuroi* species complex.

RIP Statistics	*Fusarium* Species ^1^					
	*Fc*	*Ft*	*Ff*	*Fm*	*Fv*	*Fn*
Number of RIP-affected windows	5766	5703	2741	1946	741	4468
Average RIP composite index	1.33	1.38	1.54	1.14	1.17	1.20
Average size of RIP-affected genomic regions (bp)	15,537	17,065	6542	9764	6219	12,609
Number of genes in RIP-affected regions	36	34	11	2	3	5
Number of LRARs ^2^	162	153	95	65	32	149
Gene density in LRARs ^3^	1.5 (34.41)	1.38 (31.16)	0.62 (34.42)	0.36 (31.59)	1.06 (38.93)	0.36 (30.57)
Largest LRAR (bp)	62,500	66,000	79,000	27,000	10,000	55,500
RIP-affected genomic proportion (bp)	6.39% (2,881,899)	6.30% (2,851,440)	3.13% (1,371,951)	2.10% (972,147)	0.89% (372,420)	4.32% (2,229,769)
Genomic proportion containing LRARs (bp)	5.6% (2,517,015)	5.8% (2,611,000)	1.4% (621,500)	1.4% (634,659)	0.5% (199,000)	3.6% (1,878,813)

^1^*Fc, Ft, Ff, Fm, Fv* and *Fn* refer to *F. circinatum*, *F. temperatum*, *F. fujikuroi*, *F. mangiferae*, *F. verticillioides* and *F. nygamai*, respectively. ^2^ LRAR = large RIP-affected regions (≥ 4000 bp). ^3^ Gene density is calculated per 100 kilo-base pairs.

**Table 4:** RIP statistics, GC content and gene content for the dispensable chromosomes of five fungi in the *F. fujikuroi* species complex.

Chromosome Statistics	*Fusarium* Species ^1^				
	*Fc*	*Ff*	*Ft*	*Fm*	*Fn*
Chromosome size (bp)	525,065	692,922	986,231	887,429	791,442
GC content (%)	46.36	41.05	42.30	43.02	41.7
Number of genes per chromosome	171	136	189	163	133
RIP-affected proportion (bp) ^2^	47,112	85,922	242,613	74,367	108,149
Percentage of chromosome that is RIP-affected (%) ^2^	4.85	8.23	18.25	8.38	20.66
Number of LRARs per chromosome ^3^	1	2	6	5	13
Number of genes in RIP-affected regions	0	0	1	0	0

^1^*Fc*, *Ff*, *Ft*, *Fm*, and *Fn* refer to *F. circinatum*, *F. fujikuroi*, *F. temperatum*, *F. mangiferae*, and *F. nygamai*, respectively. ^2^ Calculated using the RIP index values. ^3^ LRAR = large RIP-affected region (i.e., ≥ 4 000 bp regions of the genome experiencing RIP).

**Table 5:** RIP statistics for the genomic regions associated with AFLP markers showing transmission ratio distortion (TRD) versus those that are inherited in a Mendelian fashion ^1^.

RIP Statistics Associated with AFLP Markers		Species	
		*F. circinatum*	*F. temperatum*
TRD markers	Total number of markers	30	31
	Number of RIP-targeted markers	19	18
	Number of markers not RIP-targeted	11	13
	Proportion of the RIP-targeted total genome (%) ^2^	2.17	2.30
Mendelian markers	Total number of markers	29	26
	Number of RIP-targeted markers	9	6
	Number of markers not RIP-targeted	20	20
	Proportion of the RIP-targeted total genome (%) ^2^	0.53	1.74

^1^ AFLP = amplified fragment length polymorphism. AFLP markers previously determined [35]. TRD AFLP markers showed highly significant segregation distortion (*P* < 0.01 in the F_1_ progeny) [35], while non-TRD markers were inherited in a Mendelian fashion in a 1:1 ratio. ^2^ Determined by summing the number of windows with RIP recorded for the genomic regions that the AFLP markers mapped divided by the total count of windows with RIP detected in the genome.

## Supplementary Materials

The following are available online at https://www.mdpi.com/2076-0817/8/4/298/s1. Figure S1: Schematic illustration of RNA-seq data aligned to that of the genes identified in *Neurospora crassa* associated with RIP, to that of *Fusarium circinatum* putative homologous sequences; Figure S2: Schematic representation of the H3k9me3 markers and changes in RIP composite index values of each chromosome assembly (I–XII) of *Fusarium fujikuroi*; Figure S3: Changes in nucleotide frequencies per percentage GC content ranges; Figure S4: Summary of the genetic features of the first 12 chromosome assemblies of *Fusarium circinatum* (FSP34); Figure S5: Percentage of the core chromosomes affected by RIP, for the three different major clades Fusarium *fujikuroi* species complex; Table S1: List of RIP genes regarded to be necessary for RIP, and RIP-associated methylation to occur in RIP capable fungi; Table S2: List of InterPro terms and descriptions, and GO term and descriptions of the protein sequences that have been identified to be responsible for RIP; Table S3: Summary of the total RIP-affected windows per chromosome for each the *Fusarium fujikuroi* species complex representatives investigated; Table S4: Percentages of pairwise nucleotide identity between the chromosomes and their homologs and level of completeness as indicated by BUSCO analysis of *F. circinatum* (Fc) in *F. temperatum* (Ft), *F. fujikuroi* (Ff), *F*. *mangiferae* (Fm), *F. verticillioides* (Fv) and *F. nygamai* (Fn); Table S5: List of genomic location of large RIP-affected regions (LRARs) in core chromosomes per fungal genome investigated in this study; Table S6: Estimation of the average gene density for the genome assemblies and the large RIP-affected regions (LRARs) in the core chromosome assemblies for the FFSC species investigated in this study; Table S7: List of enriched GO terms of the LRARs in *Fusarium circinatum*; Table S8: List of AFLP markers showing highly significant transmission ratio distortion localized to the genome assemblies of *F. circinatum* and *F. temperatum*; Table S9: Genomic position, RIP status, count of RIP positive windows recorded and the composite RIP index values for the AFLP markers that were inherited according to Mendelian ratio’s localized to the genome assemblies of *Fusarium circinatum* and *Fusarium temperatum*; Table S10: List of putative pseudogenes identified in the LRAR and surrounding genomic regions *of Fusarium circinatum*; Table S11: NCBI BLASTn alignment results of the homologous genomic regions showing opposite RIP statuses of the transmission ratio distortion markers and Mendelian markers in the genome assemblies of *F. circinatum* and *F. temperatum.*
