# Supplementary material for: Repeat-Induced Point Mutations Drive Divergence between Fusarium circinatum and Its Close Relatives
Source: Pathogens. 2019 Dec 14;8(4):298. doi: 10.3390/pathogens8040298 (PMC6963459; doi:10.3390/pathogens8040298)
Supplement: Supplementary file 1 [file pathogens-08-00298-s001.zip › Figure S2 van Wyk et al 2020.pptx]

## Slide 1
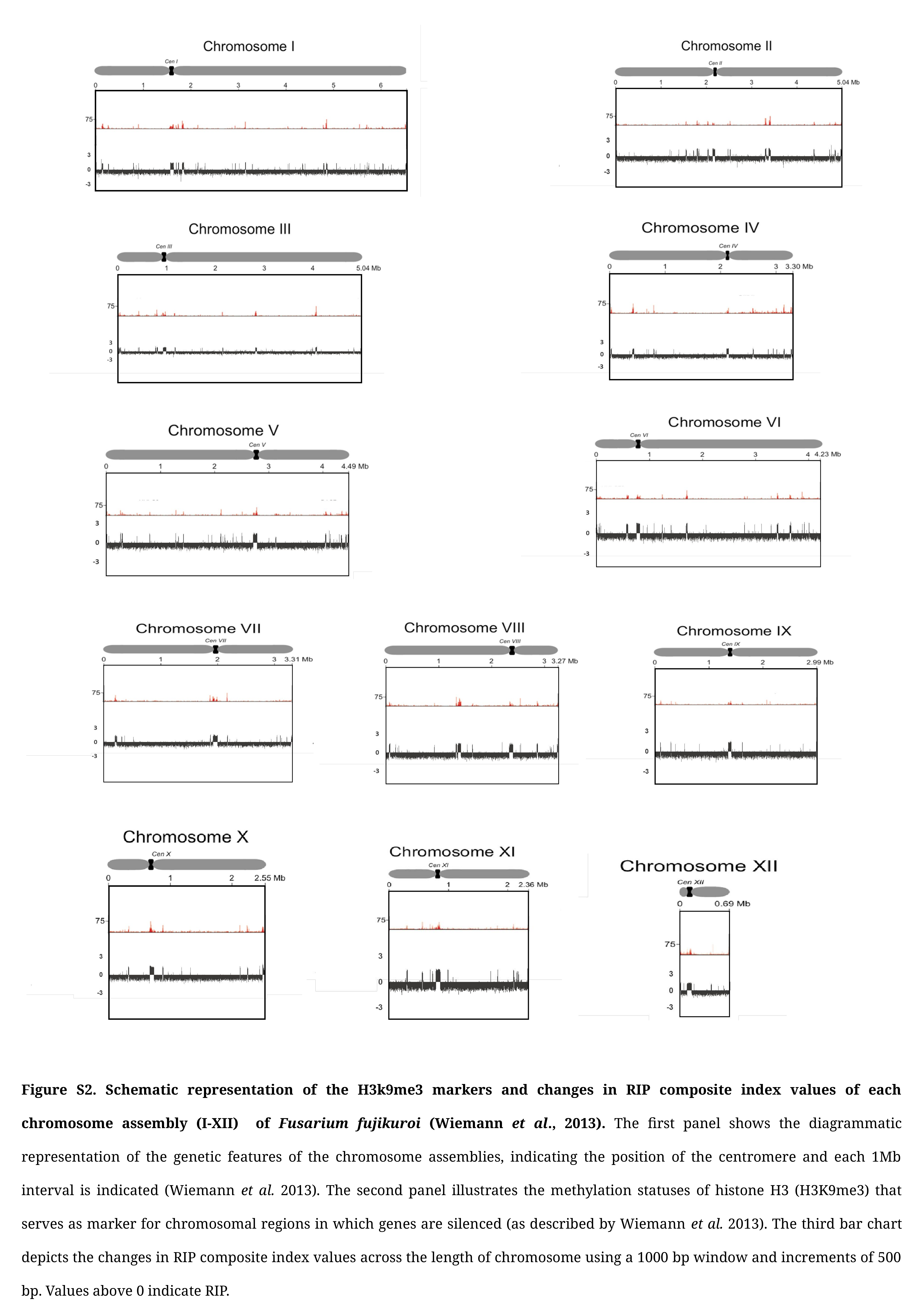

Figure S2. Schematic representation of the H3k9me3 markers and changes in RIP composite index values of each chromosome assembly (I-XII) of Fusarium fujikuroi (Wiemann et al., 2013). The first panel shows the diagrammatic representation of the genetic features of the chromosome assemblies, indicating the position of the centromere and each 1Mb interval is indicated (Wiemann et al. 2013). The second panel illustrates the methylation statuses of histone H3 (H3K9me3) that serves as marker for chromosomal regions in which genes are silenced (as described by Wiemann et al. 2013). The third bar chart depicts the changes in RIP composite index values across the length of chromosome using a 1000 bp window and increments of 500 bp. Values above 0 indicate RIP.
