# Supplementary material for: Repeat-Induced Point Mutations Drive Divergence between Fusarium circinatum and Its Close Relatives
Source: Pathogens. 2019 Dec 14;8(4):298. doi: 10.3390/pathogens8040298 (PMC6963459; doi:10.3390/pathogens8040298)
Supplement: Supplementary file 1 [file pathogens-08-00298-s001.zip › Figure S3 van Wyk et al 2020.pptx]

## Slide 1
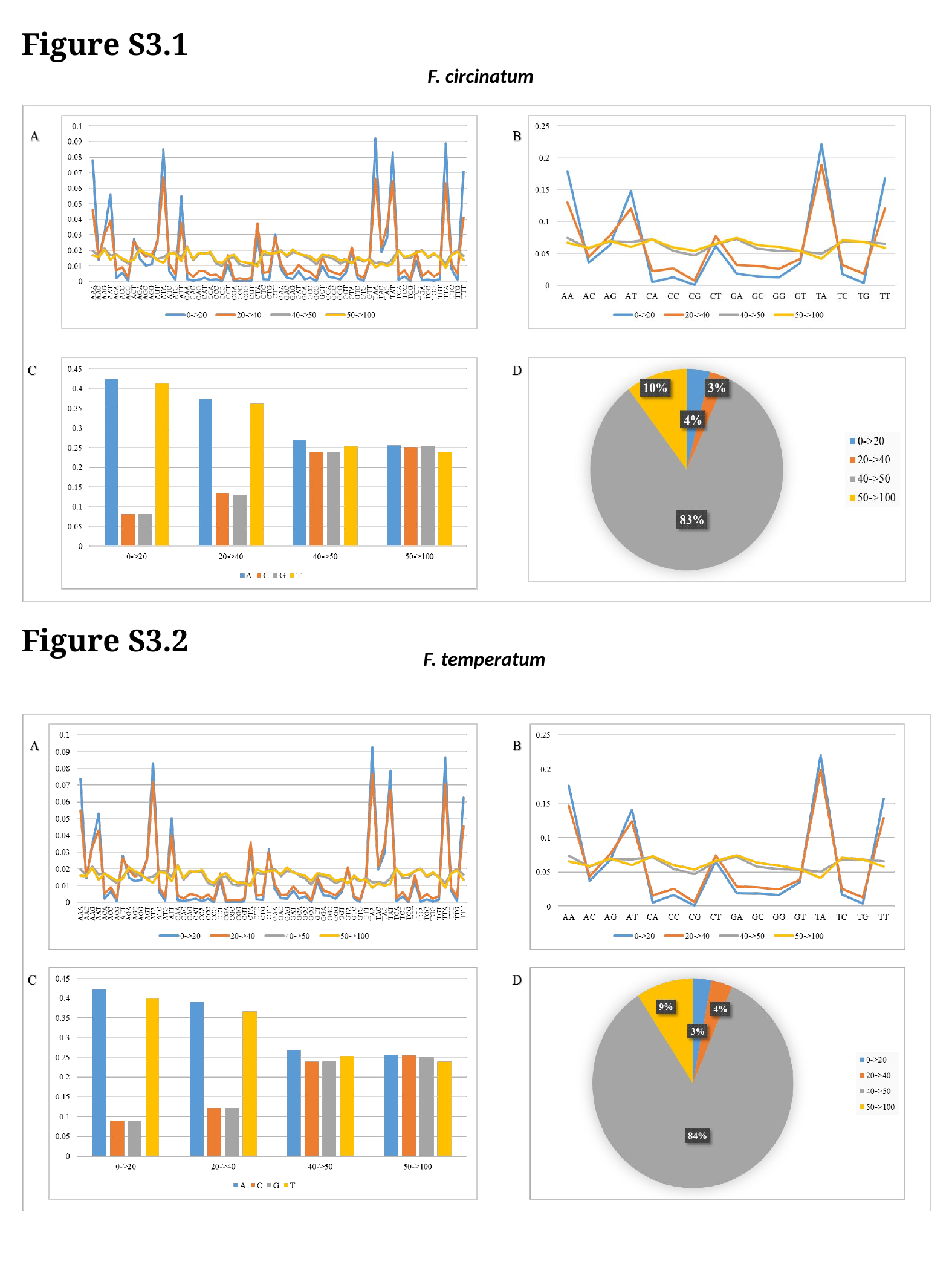

Figure S3.1
F. circinatum
Figure S3.2
F. temperatum

## Slide 2
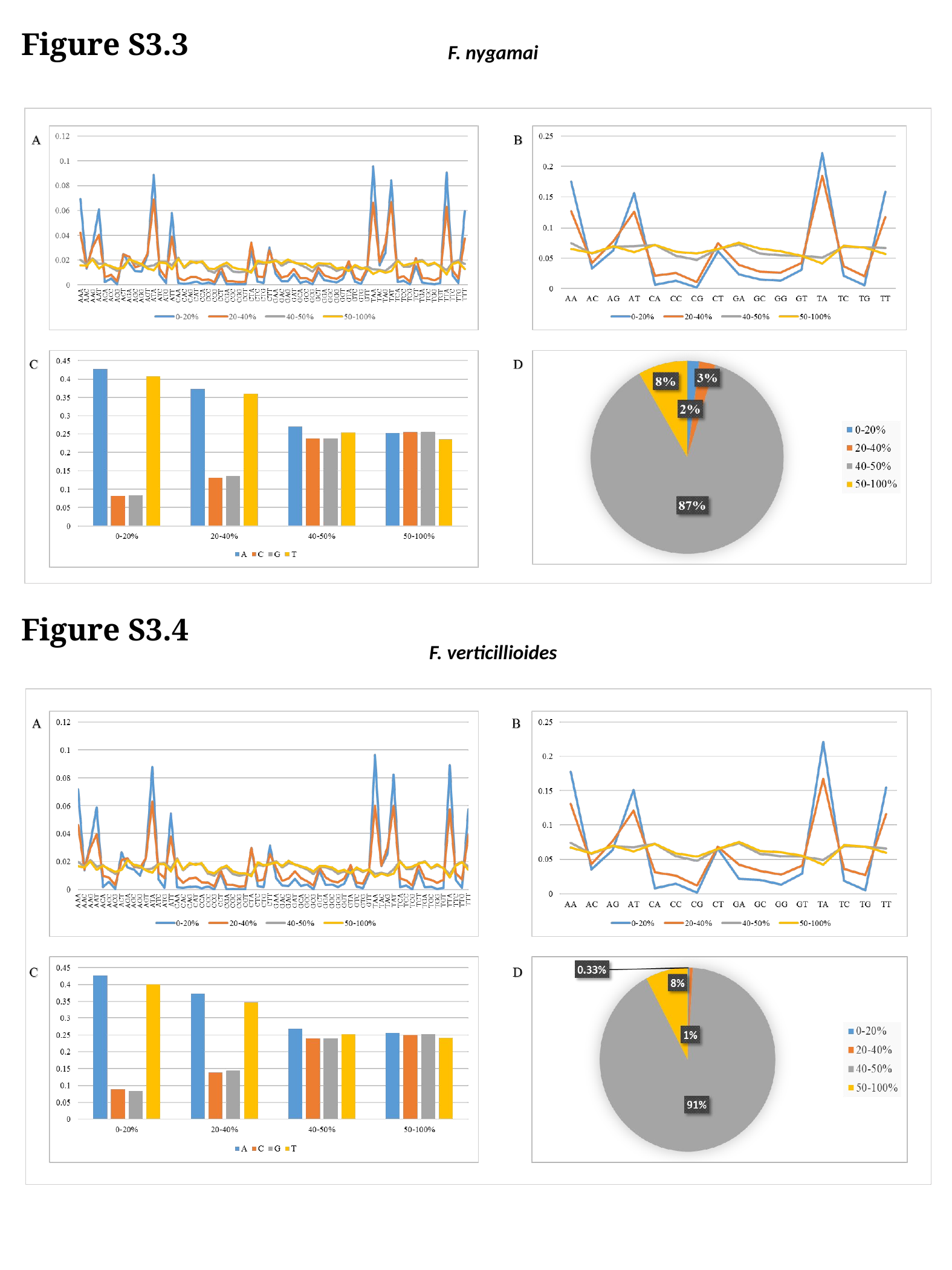

Figure S3.3
F. nygamai
Figure S3.4
F. verticillioides

## Slide 3
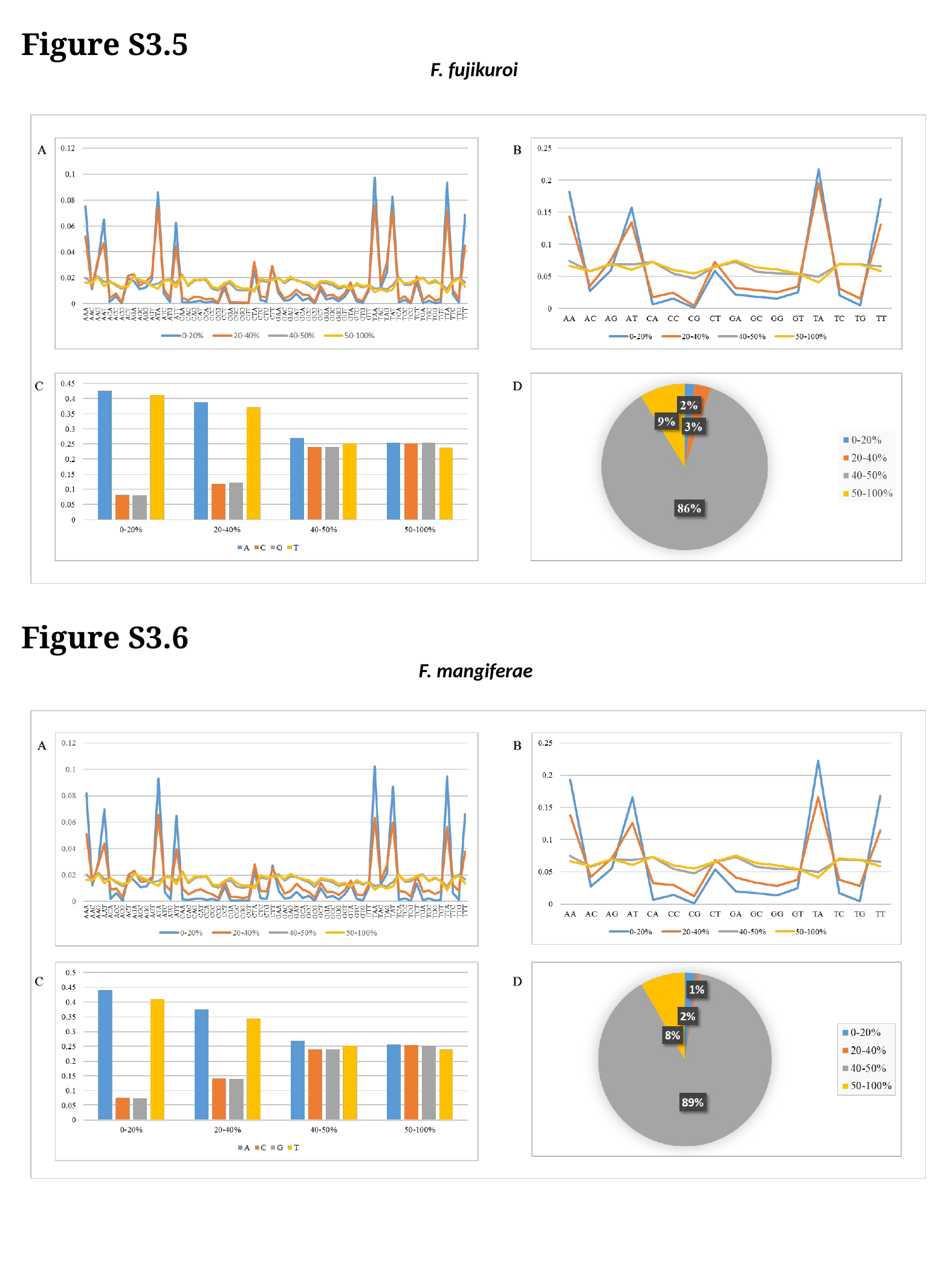

Figure S3.5
F. fujikuroi
Figure S3.6
F. mangiferae

## Slide 4
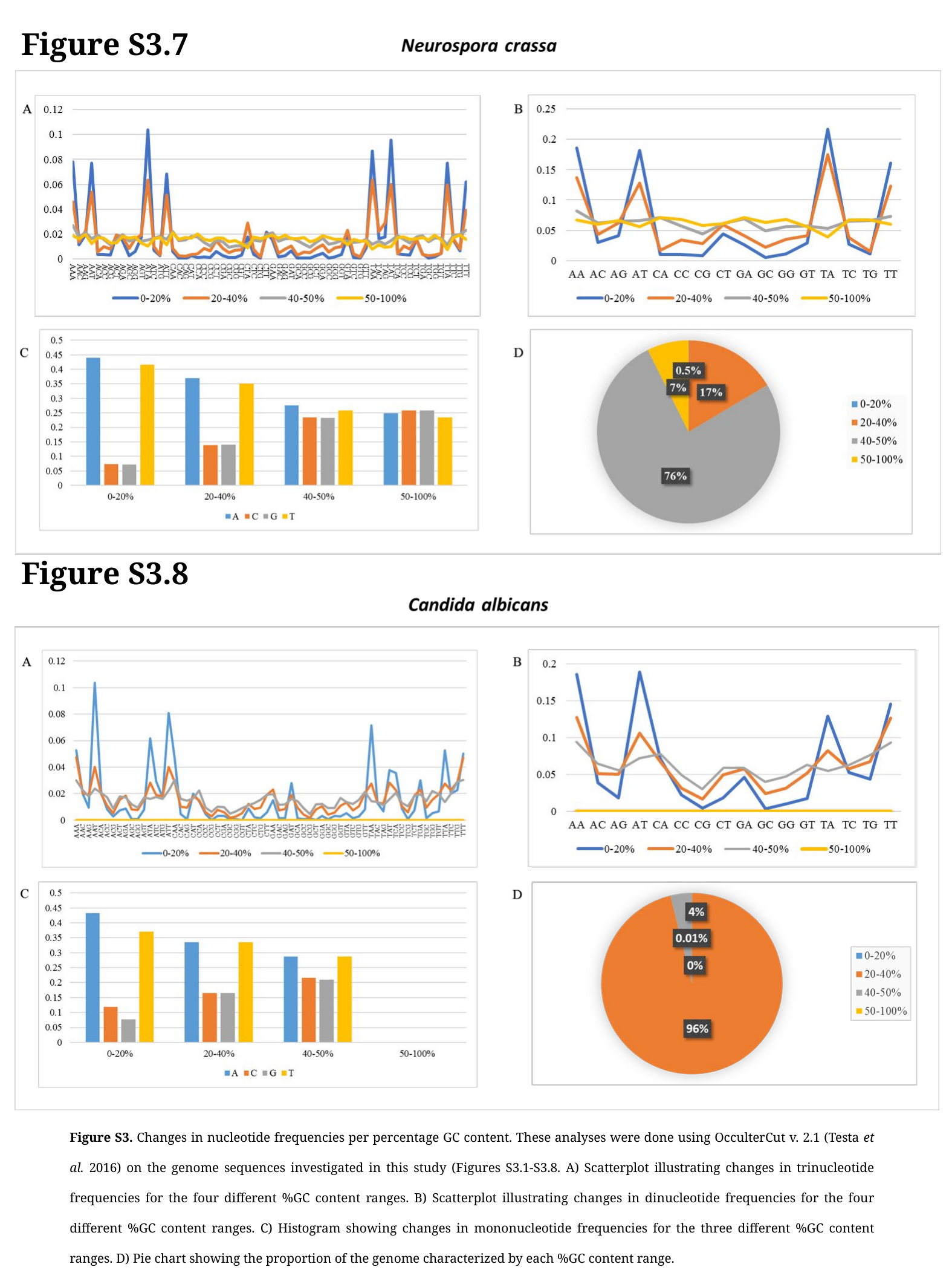

Figure S3.7
Figure S3.8
Figure S3. Changes in nucleotide frequencies per percentage GC content. These analyses were done using OcculterCut v. 2.1 (Testa et al. 2016) on the genome sequences investigated in this study (Figures S3.1-S3.8. A) Scatterplot illustrating changes in trinucleotide frequencies for the four different %GC content ranges. B) Scatterplot illustrating changes in dinucleotide frequencies for the four different %GC content ranges. C) Histogram showing changes in mononucleotide frequencies for the three different %GC content ranges. D) Pie chart showing the proportion of the genome characterized by each %GC content range.
