# Supplementary material for: Repeat-Induced Point Mutations Drive Divergence between Fusarium circinatum and Its Close Relatives
Source: Pathogens. 2019 Dec 14;8(4):298. doi: 10.3390/pathogens8040298 (PMC6963459; doi:10.3390/pathogens8040298)
Supplement: Supplementary file 1 [file pathogens-08-00298-s001.zip › Figure S4 van Wyk et al 2020.pptx]

## Slide 1
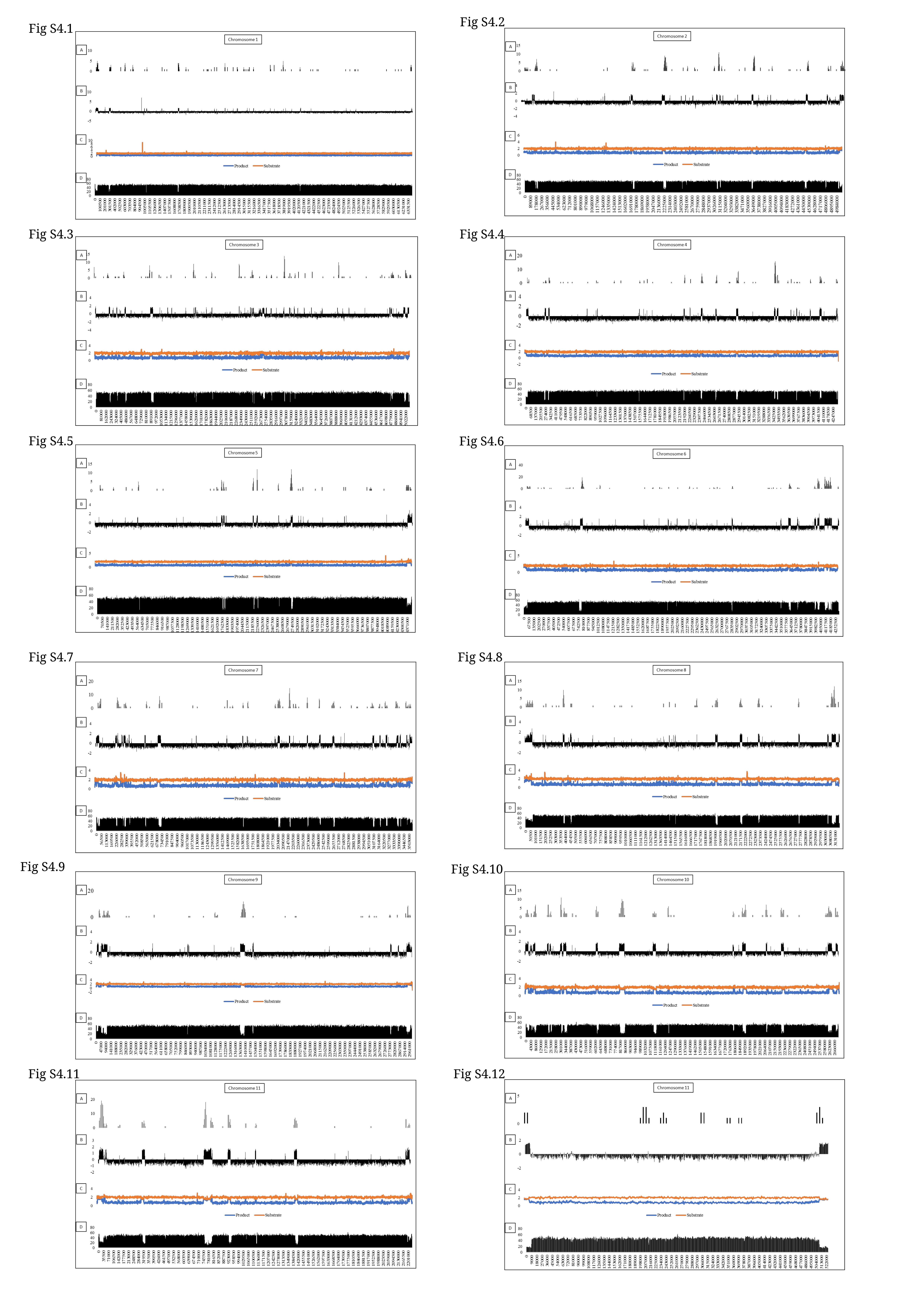

Fig S4.2
Fig S4.1
Fig S4.3
Fig S4.4
Fig S4.5
Fig S4.6
Fig S4.8
Fig S4.7
Fig S4.9
Fig S4.10
Fig S4.11
Fig S4.12

## Slide 2
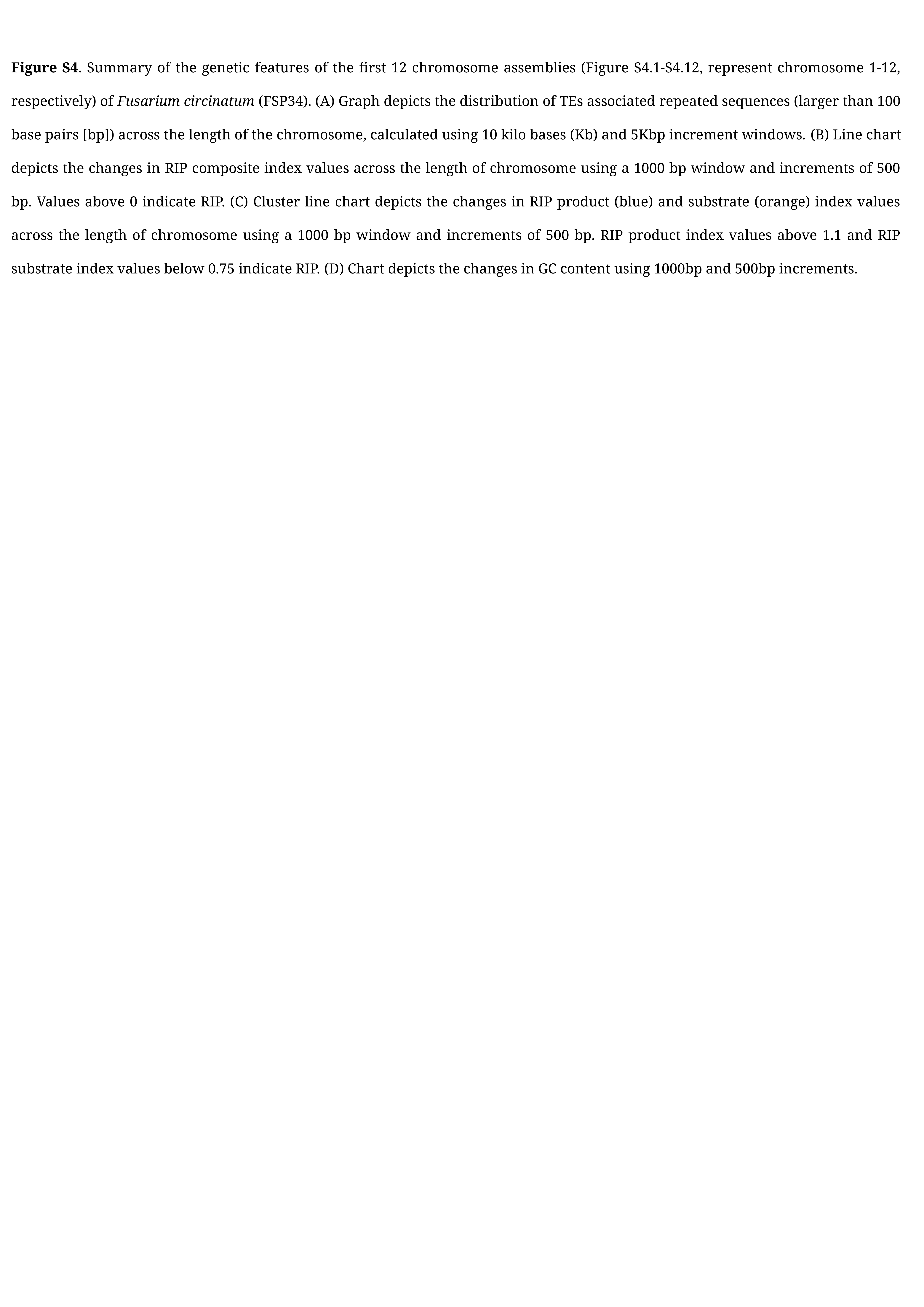

Figure S4. Summary of the genetic features of the first 12 chromosome assemblies (Figure S4.1-S4.12, represent chromosome 1-12, respectively) of Fusarium circinatum (FSP34). (A) Graph depicts the distribution of TEs associated repeated sequences (larger than 100 base pairs [bp]) across the length of the chromosome, calculated using 10 kilo bases (Kb) and 5Kbp increment windows. (B) Line chart depicts the changes in RIP composite index values across the length of chromosome using a 1000 bp window and increments of 500 bp. Values above 0 indicate RIP. (C) Cluster line chart depicts the changes in RIP product (blue) and substrate (orange) index values across the length of chromosome using a 1000 bp window and increments of 500 bp. RIP product index values above 1.1 and RIP substrate index values below 0.75 indicate RIP. (D) Chart depicts the changes in GC content using 1000bp and 500bp increments.
