# Supplementary material for: Repeat-Induced Point Mutations Drive Divergence between Fusarium circinatum and Its Close Relatives
Source: Pathogens. 2019 Dec 14;8(4):298. doi: 10.3390/pathogens8040298 (PMC6963459; doi:10.3390/pathogens8040298)
Supplement: Supplementary file 1 [file pathogens-08-00298-s001.zip › Figure S5 van Wyk et al 2020.pptx]

## Slide 1
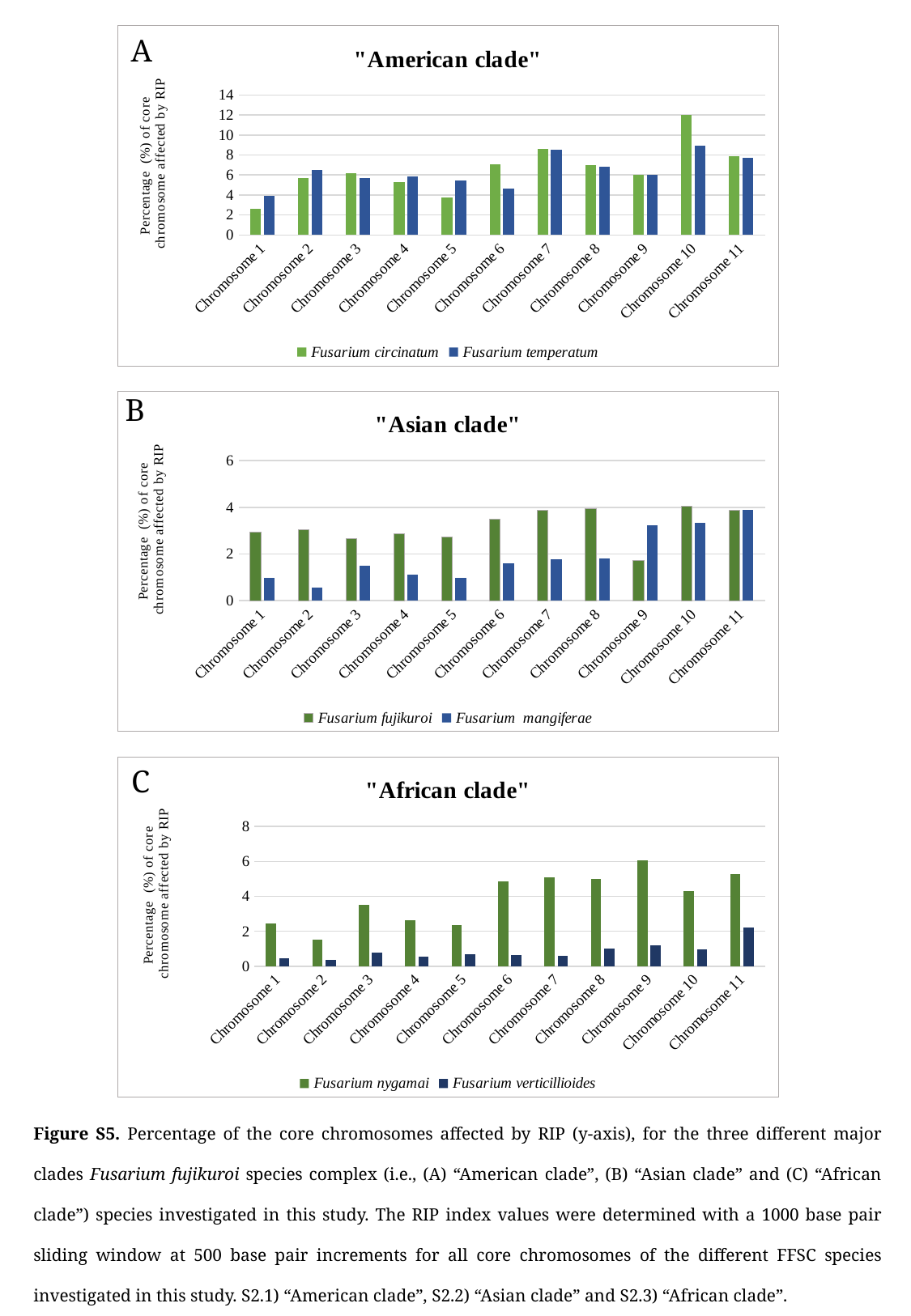

A
### Chart: "American clade"
| Category | Fusarium circinatum | Fusarium temperatum |
|---|---|---|
| Chromosome 1 | 2.65 | 3.88 |
| Chromosome 2 | 5.71 | 6.47 |
| Chromosome 3 | 6.18 | 5.66 |
| Chromosome 4 | 5.3 | 5.86 |
| Chromosome 5 | 3.71 | 5.49 |
| Chromosome 6 | 7.11 | 4.62 |
| Chromosome 7 | 8.6 | 8.51 |
| Chromosome 8 | 6.97 | 6.86 |
| Chromosome 9 | 6.05 | 6.04 |
| Chromosome 10 | 11.99 | 8.97 |
| Chromosome 11 | 7.92 | 7.76 |B
### Chart: "Asian clade"
| Category | Fusarium fujikuroi | Fusarium mangiferae |
|---|---|---|
| Chromosome 1 | 2.92 | 0.99 |
| Chromosome 2 | 3.04 | 0.55 |
| Chromosome 3 | 2.66 | 1.5 |
| Chromosome 4 | 2.86 | 1.11 |
| Chromosome 5 | 2.72 | 0.99 |
| Chromosome 6 | 3.49 | 1.61 |
| Chromosome 7 | 3.87 | 1.79 |
| Chromosome 8 | 3.95 | 1.81 |
| Chromosome 9 | 1.73 | 3.23 |
| Chromosome 10 | 4.05 | 3.35 |
| Chromosome 11 | 3.88 | 3.89 |
### Chart: "African clade"
| Category | Fusarium nygamai | Fusarium verticillioides |
|---|---|---|
| Chromosome 1 | 2.46 | 0.47 |
| Chromosome 2 | 1.5 | 0.35 |
| Chromosome 3 | 3.53 | 0.79 |
| Chromosome 4 | 2.66 | 0.54 |
| Chromosome 5 | 2.37 | 0.69 |
| Chromosome 6 | 4.87 | 0.63 |
| Chromosome 7 | 5.09 | 0.6 |
| Chromosome 8 | 5.01 | 1.01 |
| Chromosome 9 | 6.08 | 1.18 |
| Chromosome 10 | 4.32 | 0.95 |
| Chromosome 11 | 5.28 | 2.23 |C
Figure S5. Percentage of the core chromosomes affected by RIP (y-axis), for the three different major clades Fusarium fujikuroi species complex (i.e., (A) “American clade”, (B) “Asian clade” and (C) “African clade”) species investigated in this study. The RIP index values were determined with a 1000 base pair sliding window at 500 base pair increments for all core chromosomes of the different FFSC species investigated in this study. S2.1) “American clade”, S2.2) “Asian clade” and S2.3) “African clade”.
