# Supplementary material for: Repeat-Induced Point Mutations Drive Divergence between Fusarium circinatum and Its Close Relatives
Source: Pathogens. 2019 Dec 14;8(4):298. doi: 10.3390/pathogens8040298 (PMC6963459; doi:10.3390/pathogens8040298)
Supplement: Supplementary file 1 [file pathogens-08-00298-s001.zip › Figure S1 van Wyk et al 2020.pptx]

## Slide 1
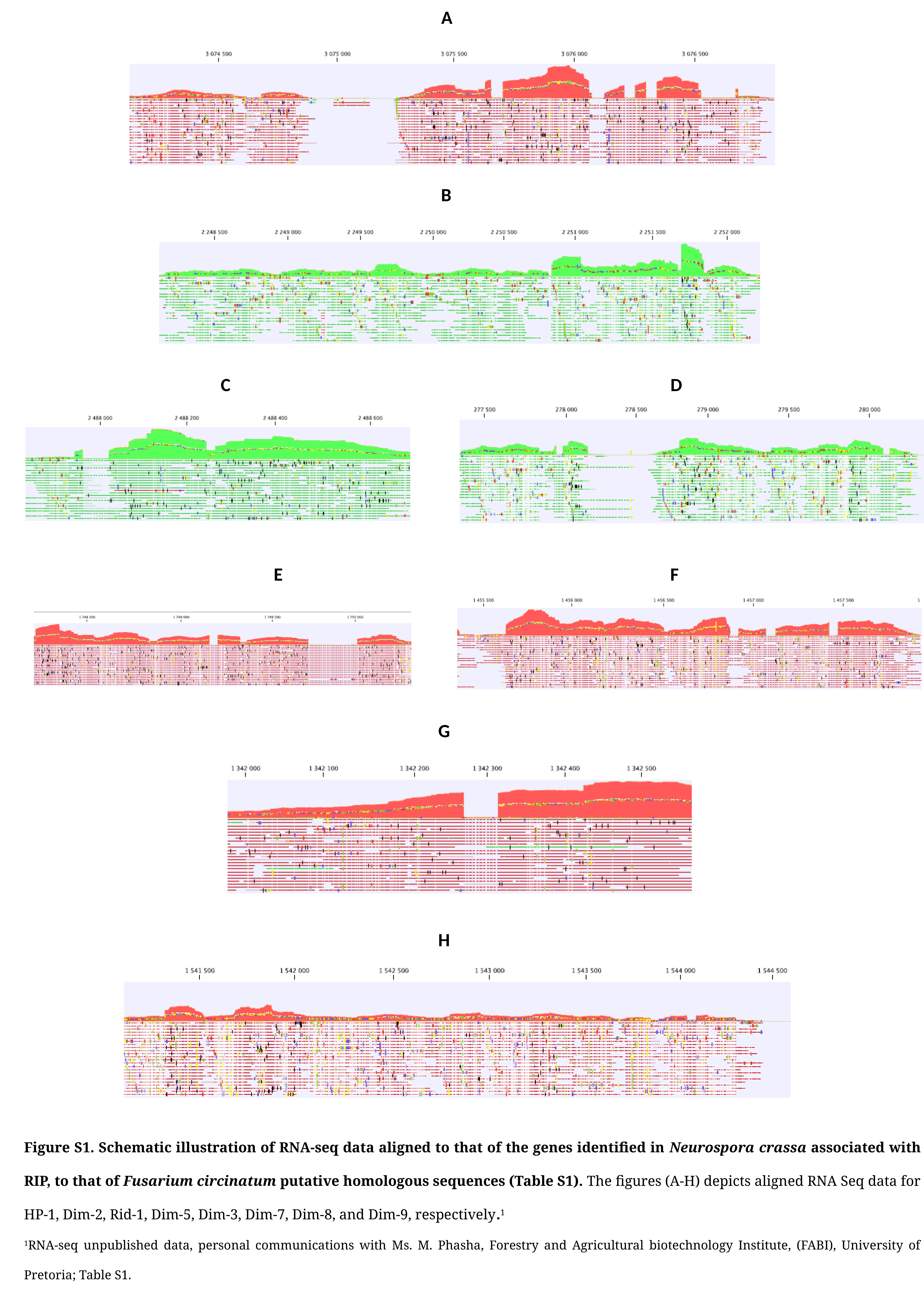

A
B
C
D
E
F
G
H
Figure S1. Schematic illustration of RNA-seq data aligned to that of the genes identified in Neurospora crassa associated with RIP, to that of Fusarium circinatum putative homologous sequences (Table S1). The figures (A-H) depicts aligned RNA Seq data for HP-1, Dim-2, Rid-1, Dim-5, Dim-3, Dim-7, Dim-8, and Dim-9, respectively.1
1RNA-seq unpublished data, personal communications with Ms. M. Phasha, Forestry and Agricultural biotechnology Institute, (FABI), University of Pretoria; Table S1.
